# Genomic analysis reveals independent evolution of *Plasmodium falciparum* populations in Ethiopia

**DOI:** 10.1186/s12936-021-03660-y

**Published:** 2021-03-04

**Authors:** Deriba Abera, Caleb K. Kibet, Teshome Degefa, Lucas Amenga-Etego, Joel L. Bargul, Lemu Golassa

**Affiliations:** 1 Department of Biochemistry, College of Health Sciences, Jomo Kenyatta University of Agriculture and Technology, Nairobi, Kenya; 2 School of Medical Laboratory Sciences, Faculty of Health Sciences, Jimma University, Jimma, Ethiopia; 3 International Centre of Insect Physiology and Ecology, Nairobi, Kenya; 4 West African Centre for Cell Biology of Infectious Pathogens (WACCBIP), University of Ghana, Accra, Ghana; 5 Aklilu Lemma Institute of Pathobiology, Addis Ababa University, Addis Ababa, Ethiopia

**Keywords:** *Plasmodium falciparum*, Ethiopia, Population structure, Drug resistance, Admixture, Positive selection

## Abstract

**Background:**

*Plasmodium falciparum* parasite populations in Ethiopia have been experiencing local selective pressures from drugs and immunity, leading to evolutionary adaptation. However, there was a paucity of data on genomic characterization and evolutionary adaptations of *P. falciparum* isolates from the central area of Ethiopia.

**Methods:**

Whole-genome analysis of 25 *P. falciparum* isolates from central Ethiopia, specifically from West Arsi, were studied to determine their genetic diversity, population structures, and signatures of selection in known drug resistance alleles against global isolates from Cambodia, Thailand, DR Congo, and Malawi.

**Results:**

A total of 18,517 high-quality single-nucleotide polymorphisms (SNPs) were identified in Ethiopian *P. falciparum* isolates. About 84% of the Ethiopian *P. falciparum* isolates had a F_WS_ value > 0.95 showing a dominant single genotype infection in most isolates at the time of collection with little potential for out-crossing as expected in areas with low transmission intensity. Within-host diversity of Ethiopian infections was significantly different from East African (p < 0.001), but not Southeast Asian infections (P > 0.05). A significant population structure has been observed by PCA and population differentiation between Ethiopian parasites and East African (Fst ~ 10%) and Southeast Asian populations (Fst ~ 18%), suggesting limited gene flow and the independent evolution of the Ethiopian parasite population. Moreover, a total of 125 genes under balancing selection was found that include *ama1*, *trap*, *eba175*, and *lsa3*, previously identified as targets of human host immunity. Recent directional selection analysis using integrated standardized haplotype score (IHS) did not detect any selection signatures in the *Pfcrt*, *Pfdhfr*, *Pfdhps*, *Pfmdr*1, and *PfK13* genes. However, known drug resistance-conferring mutations analysis showed that at least one SNP marker was fixed in these genes, but not in *Pfdhps* and *PfK13*.

**Conclusion:**

*Plasmodium falciparum* populations in the central region of Ethiopia was structurally diverged from both Southeast Asian and other East African populations. Malaria infections in Ethiopia had low within-host diversity, and parasites carry fixed chloroquine resistance markers despite the withdrawal of this drug for the treatment of *P. falciparum*.

**Supplementary Information:**

The online version contains supplementary material available at 10.1186/s12936-021-03660-y.

## Background

*Plasmodium falciparum* malaria remains one of the major public health problems worldwide accounting for 228 million cases in 2018 compared to 231 million in 2017, while the number of deaths due to malaria decreased by just 2.5%, from 415,000 to 405,000 during the same period [1]. Sub-Saharan Africa (sSA) still accounts for 94% of global death. In Ethiopia, more than 75% of the total area is malarious, and *P. falciparum* and *Plasmodium vivax* co-exist [2] making malaria control more complicated than in other African countries.

Across malaria-endemic regions, large-scale deployment of anti-malarial drugs has led to the emergence of drug resistance to chloroquine (CQ) and sulfadoxine/pyrimethamine (SP) antifolate drugs [3–5]. Like many other countries, Ethiopia has switched from CQ to SP in 1998 and from SP to artemether–lumefantrine (AL) in 2004 [6] for the treatment of uncomplicated *P. falciparum* malaria in response to the development of parasite resistance. However, CQ remained the first-line drug for *P. vivax* treatment in the country [7], leading to a continued selection of CQ-resistance markers in *P. falciparum* as the result of indirect pressure from CQ and the presence of mixed infections of *P. falciparum* and *P. vivax*. Similar to CQ and SP, *P. falciparum* developed resistance to AL first at the Thai-Cambodian border [8] and recently in East Africa [9]. The rapid development of resistance in *P. falciparum* to series of the first-line anti-malarial hinders malaria prevention, control, and elimination efforts.

Anti-malarial drugs are known to pose tremendous selective pressure on *P. falciparum* leading to the worldwide spread of resistant parasites [10]. It was well noted that *P. falciparum* resistance to the two conventional anti-malarial drugs, CQ and SP, has resulted in increased malaria morbidity and mortality across endemic settings. Apart from increased morbidity and mortality, selective sweeps of drug resistance mutations have reduced levels of polymorphism in *P. falciparum* as these resistant and sensitive strains continue to recombine in mosquitoes [11, 12], with perhaps a reduced diversity around the selected loci. However, the greatly reduced level of diversity across the entire *P. falciparum* genome most likely resulted from a severe population bottleneck as has been observed in gorilla-to-human cross-species transmission [13].

Based on the analysis made on 12 strains collected from different countries in Africa and Asia, the average diversity of *P. falciparum* at fourfold degenerate sites was estimated to be 8 × 10^−4^ per site [13]. However, published mutation rates for *P. falciparum* were in the range of 1–10 × 10^−9^ mutations per site per replication cycle [14, 15]. Depending on *P. falciparum* life cycle and assuming varying lengths of time that the parasites spend either in the vector or in the mammalian host, the parasites are likely to undergo at least 200 replication cycles per year suggesting that the observed level of genetic diversity in *P. falciparum* could have readily accumulated within the past 10,000 years [10].

*Plasmodium falciparum* parasite change and select its new genetic variants owing to drug exposure to cause disease and overcome challenges from host immunity and therapeutic interventions [16]. Indeed, high pressure from immunity and drugs are known to select adaptive parasite strains that maintain transmission [17] and, therefore, many *P. falciparum* genes encoding immune and drug targets are under natural selection and show signatures of balancing or directional selection [4, 17–21]. This selection may vary due to differences in innate susceptibility of human populations, variations in ecological transmission, resulting in varying degrees of acquired immunity and/or drug pressure. Malaria parasites from low and high endemic regions have a distinct opportunity for transmission and host acquired immune responses [22]. For effective management of malaria control and intervention strategies, it is important to determine genetic variation patterns due to parasite adaptation to host environments and drug interventions. Balancing selection brings the favoured alleles of parasites to an intermediate equilibrium where they are maintained as genetic polymorphisms, while the directional selection forces cause the parasite’s genetic variants to increase in frequency and facilitate the occurrence of selective sweeps around the affected loci [23].

*Plasmodium falciparum* population genomics has been highly studied in West African populations and showed signatures of balancing selection on multiple candidate vaccine antigens and strong directional selection around known drug resistance genes [19, 24]. In contrast, there is little information about the genomic variations of *P. falciparum* populations in the horn of Africa, including Ethiopia, where *P. falciparum* and *P. vivax* malaria co-exist and are heterogeneously distributed. A recent study reported *P. falciparum* populations in the horn of Africa, specifically in Ethiopia, are unique and structurally diverged from other West, East, and central African *P. falciparum* populations [25]. These parasite populations share a chunk of genes with other sub-Saharan African *P. falciparum* populations across drug and immune targets and facilitate the spread of drug-resistant strains [25]. These studies call for in-depth analysis of Ethiopian parasite genomes to deepen understanding of genome diversity and natural selection in Ethiopia’s unique human populations with co-species transmission dynamics.

Understanding the population genetic diversity of *P. falciparum* strains circulating in the specific region of central Ethiopia is very important to monitor the effectiveness of control schemes and provide baseline information for making informed decisions by the national malaria control programme [26]. This study aimed to characterize the *P. falciparum* populations in West Arsi, in central Ethiopia, using whole-genome analysis of data generated by Illumina next-generation sequencing.

## Methods

### Study area and population

The study was conducted in West Arsi, Oromia (07′ 17′′ 34.2 S, 038′ 21″ 46.3 W) located about 251 km from Addis Ababa, Ethiopia. This region with distinct wet and dry seasons has an altitude of about 1500–2300 m above sea level with the human population of 176,671. The inhabitants of this malarious region have high levels of poverty worsened by the malaria diseases caused predominantly by *P. falciparum* and *P. vivax* with a seasonal and unstable pattern of transmission [7].

### Sample collection and processing

Venous blood (2–5 mL) was collected from July 2012 to December 2013 from consented *P. falciparum* malaria patients following standard procedures. Sequencing of 34 *P. falciparum* samples from leukocyte-depleted infected whole blood was done as described by Auburn et al*.* [27], at the Welcome Sanger Institute as part of the MalariaGEN *P. falciparum* Community Project (www.malariagen.net/projects). Freely available *P. falciparum* sequence data were accessed via the Pf3K project (https://www.malariagen.net/data/pf3k-5) for Southeast Asian and East African samples. Among East Africa, samples are available only in DR Congo and Malawi from East Africa and randomly took Cambodia and Thailand. Sample collection site with the number of samples greater or equal to Ethiopian samples with closer/similar *P. falciparum* samples collection year to Ethiopian sample were randomly selected. Then, 50 samples were randomly selected from each country.

Short sequence reads were generated on the IlluminaHiSeq platform and aligned to Pf3D7 reference (version 3) by burrows-wheeler-aligners (BWA). SNP calling was done following a customized genome analysis tool kit (GATK) pipeline. Each sample was genotyped for polymorphic coding SNPs across the genome, ensuring a minimum of 5× paired-end coverage across each variant per sample. Polymorphic sites within hyper-variable, telomeric, and repetitive sequence regions were excluded. Biallelic high-quality SNPs with mapping quality (MQ) > 20 and Variant Quality Score (VQSLOD) ≥ 3 in the core region loci with a minor allele frequency of at least 2% and individual sample with less than 10% missing data and SNP-site missing less than 10% across the isolate was extracted and used for downstream analysis. After quality filtering, 46, 50, 25, 50, 49 samples of Cambodia, DR Congo, Ethiopia, Malawi, and Thailand were left, respectively.

### Analysis of population genetic diversity and within-host infection diversity

The genome-wide F_WS_ (inbreeding coefficient within a population) metric was used to calculate within-host diversity as described by Manske et al*.* [21]. To derive F_WS_ (= 1 − H_W_/H_S_), within isolate, expected heterozygosity (H_W_) was calculated from the relative allele frequencies for all genic SNPs, averaged across the genome and compared with the heterozygosity of local population (H_S_). F_WS_ value ranged from zero to one, where zero indicates high diversity of infection, and one represents a single infection within the sample as compared to local population diversity. For this analysis, individual alleles with coverage of less than < 5 reads and positions with total coverage of < 20 reads were classified as undermined (missing). Isolates with greater than > 10% missing SNP data and SNPs with > 10% missing isolate data were discarded. Isolates with F_WS_ scores of > 0.95 were classified as single predominant genotype infections.

### Population structure and admixture analysis

Principal component analysis (PCA) was used to estimate population structure using the *glPCA* function in the open-source R statistical software version 3.6.2. The first 10 principal components axis (PCs) were calculated and the first three PCs which explained the majority of the variation in the data were retained. The data was thinned downed by pruning SNPs with pairwise linkage disequilibrium (LD) by r^2^ greater than 0.05 for determining the PCs. The pruned SNP loci employed in the *glPCA* function was used to calculate an allele sharing matrix in custom Rscripts. This function use variance between and within groups to determine population genetic structure. A discriminant analysis of principal components (DAPC) [28] was used to transform the PCA data, and perform discriminant analysis on the retained principal components using the *adegenet* package in the *R* software version 3.6.2. Population admixture was determined based on spatial modelling of allele sharing among geographical coordinates of sampling sites. DAPC determines ancestry proportions and membership probability modelled on genetic variation across space to determine admixtures as described by Jombart et al. [28].

### Allele frequency and differentiation analysis

Analyses of allele frequency distributions between-population F_ST_ values [29] were calculated using Vcftools or *hierfstat* package from *adegenet* in Rafter excluding SNPs with greater than 10% missing data. For F_ST_ analysis, missing data were excluded on the SNP basis with the size of each population corrected to account for F_ST_ value difference due to population size variation.

### Detection of signatures of natural selection

Within-population Tajima’s D index [30] was calculated using Vcf tools. Tajima D values were determined for each SNP and the average value for each gene was calculated. Genes with at least five SNP and positive Tajima D values > 1 were considered as genes under balancing selection.

The standardized integrated haplotype score (IHS) analysis was used to identify positive directional selection signatures by using phased SNP data with allele frequency > 5%. IHS was determined using the *rehh* package in R software with default parameters [31] after imputing missing SNP data using Beagle version 5.2. The |IHS| > 2.5 (top 1% of the expected distribution) was used as cut off value [32] to report genes under recent directional selection as reported for genome analysis of West African *P. falciparum* [17].

## Results

### Sequencing of *P. falciparum* and analysis of allele frequency

High-quality sequence data obtained from 25 *P. falciparum* clinical isolates (Additional file 1) collected from the West Arsi of Ethiopia enabled the identification of 672,956 biallelic SNPs with less than 10% missing SNPs data and < 10% sample missing data in the individual isolate. All isolates had 95.95% (645,715/672,956) SNPs call. Sequences from the intergenic regions had lower read coverage compared to those sequences in the coding regions, and as a result, 78.92% (531,120/672,956) of all SNPs called were located within genes. Of 5058 genes analysed, 3370 genes had at least one SNP (Table 1, Additional file 2: Fig. S1A). About 18,517 SNPs were polymorphic marker in at least one sample in Ethiopian (n = 25) samples of which 43.4% (8037/18,517) were non-synonymous coding SNPs, 22.8% (4222/18,517) synonymous coding SNPs, 26.6% (4932/18,517) in intergenic regions, 3.6% (666/18,517) other intragenic regions and 3.5% (656/18,517) SNPs in intron region. Similarly, *P. falciparum* populations from Cambodia (n = 46), DR Congo (n = 50), Malawi (n = 50), and Thailand (n = 49) had 32,854, 68,476, 79,250 and 30,427 biallelic polymorphic SNPs marker in at least one sample, respectively (Table 1, Additional file 2: Fig. S1B). The proportion of non-synonymous coding to synonymous coding and the intragenic to intergenic SNPs were ~ 2 or above in all populations (Table 1).

**Table 1:** Distribution of polymorphic SNP marker effects and their relative proportion in each *P. falciparum* population

Country of origin	Non-synonymous coding	Synonymous coding	Intergenic	Intron	Other intragenic	Proportion of non-synonymous to synonymous coding	Proportion of intragenic to intergenic	Total
SNP in Cambodia	13,271	6063	11,059	1494	806	2.19	1.96	32,693
SNP in DR Congo	29,762	14,450	19,685	3034	1255	2.06	2.46	68,186
SNP in Ethiopia	8037	4226	4932	656	666	1.90	2.75	18,517
SNP in Malawi	31,756	15,623	26,538	3608	1384	2.03	1.97	78,909
SNP in Thailand	12,194	5489	10,466	1342	792	2.22	1.89	30,283

In general, all populations had a high percentage of non-synonymous coding SNPs at polymorphic marker consistent with previous findings [17]. SNPs with minor allele frequency (MAF) < 5% were common in all analysed *P. falciparum* populations following the exclusion of monomorphic SNPs in each population. Further, SNPs with minor allele frequency of < 5% occurred more frequently in samples from Malawi than in Ethiopian isolates (Additional file 2: Fig. S2).

### Genomic diversity of *P. falciparum* infections

**Fig. 1:**
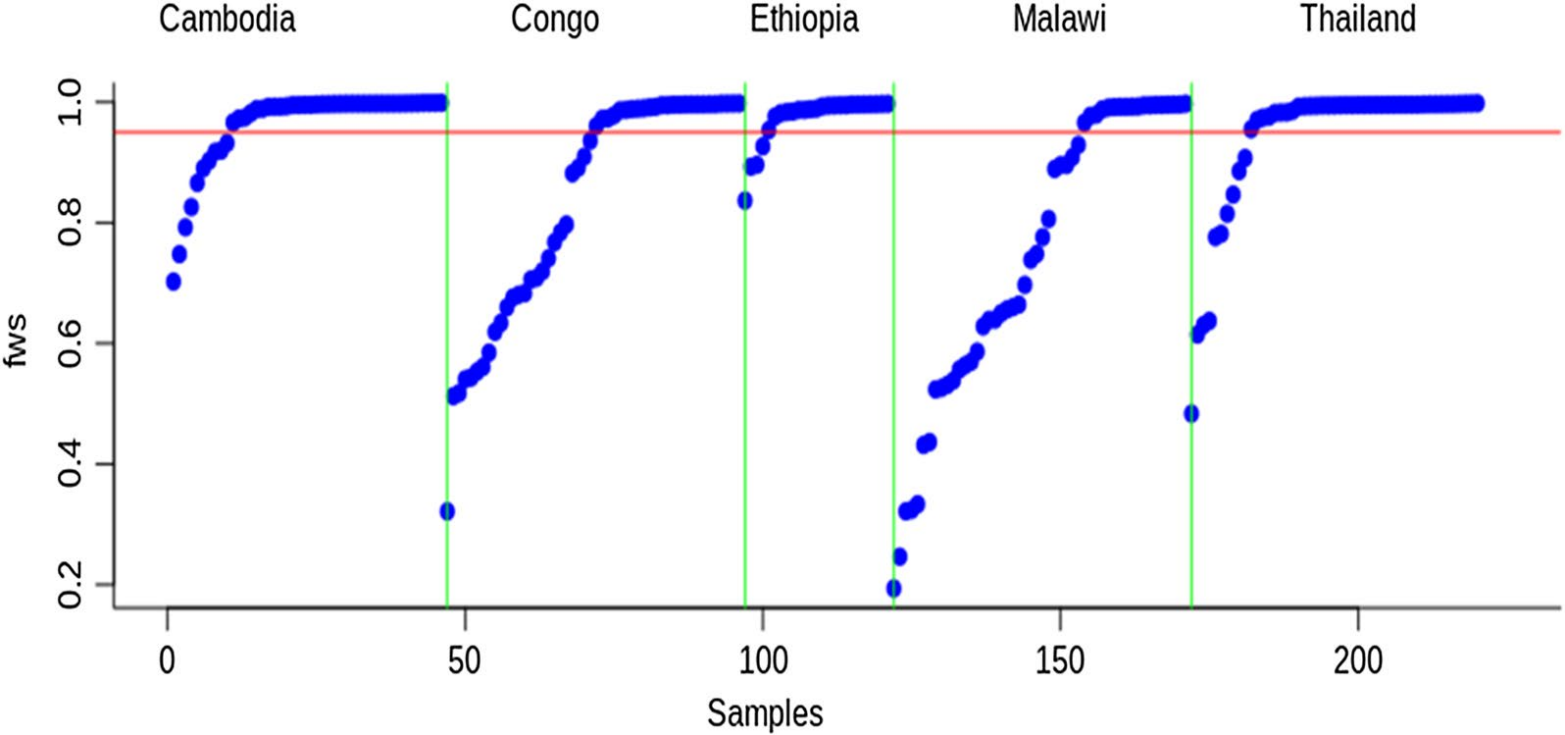
Ranked distribution of F_WS_ values by population. Redline marks F_WS_ = 0.95, above which an infection is considered clonal. A vertical green line separates the *P. falciparum* population

F_WS_ scores ranged from 0.837 to 0.997 (mean = 0.97, median = 0.99) for Ethiopian *P. falciparum* infections whereas the F_WS_ values in Cambodia ranged from 0.702 to 0.999 (mean = 0.962, median = 0.995), from 0.483 to 0.998 (mean = 0.94, median = 0.994) in Thailand, from 0.321 to 0.998 (mean = 0.94, median = 0.994) in DR Congo and from 0.194 to 0.997 (mean = 0.747, median = 0.762) in Malawi (Fig. 1; Additional file 3).

The F_WS_ value of > 0.95 suggests that the individual samples predominantly contained a single genotype and could have other additional genotypes in lower proportions. In this study, F_WS_ values of > 0.95 were observed in 84%, 79.6%, 78%, 50%, and 36% of samples from Ethiopia, Thailand, Cambodia, DR Congo, and Malawi, respectively.

The mean F_WS_ scores of the Ethiopian *P. falciparum* population were not significantly different from Cambodia’s (Welch two Sample t-test, P = 0.42) and Thailand’s (Welch two-sample t-test, p = 0.083) at 95% confidence intervals. However, mean F_WS_ was significantly higher in Ethiopia compared to DR Congo (Welch two-sample t-test, p = 5.603e^−06^) and Malawi (Welch two-sample t-test, p = 3.242e^−08^) at 95% confidence intervals.

### Population structure and admixtures

**Fig. 2:**
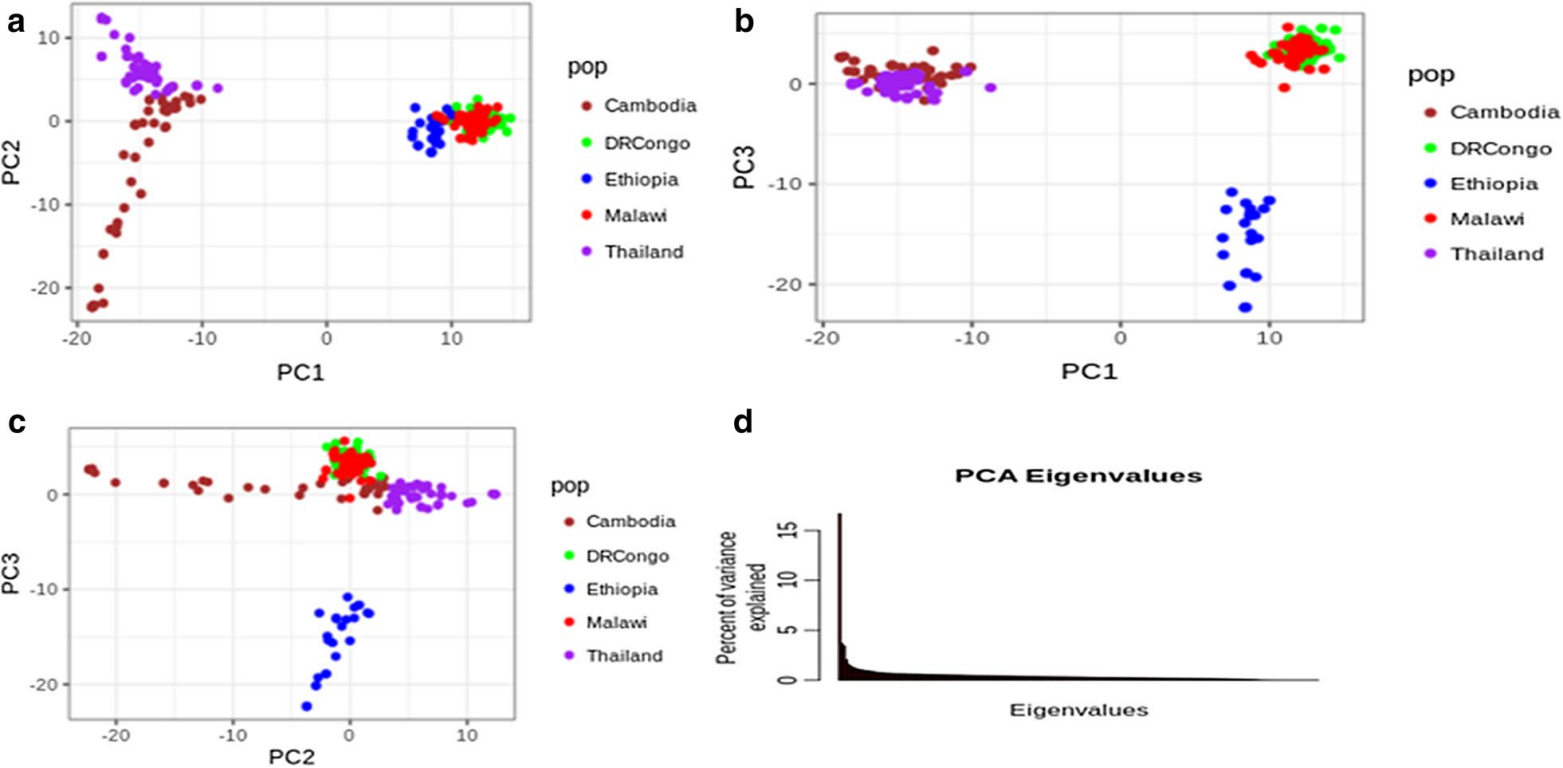
Principal component analysis. **a** Principal component axis1 and axis2 identified Southeast Asian from African *P. falciparum* population. **b** Principal component axis1 and axis3 identified Ethiopian, Southeast Asian and other East African *P. falciparum* populations. **c** Principal Component axis2 and axis3 identified Ethiopian *P. falciparum* population from Southeast Asian and African *P. falciparum* population. **d** Percent of variance explained by each principal component axis. PC1 explained 16.7%, PC2 explained 3.7% and PC3 explained 3.4% of the variance in the data. Pop stands for the *P. falciparum* country of origin

**Fig. 3:**
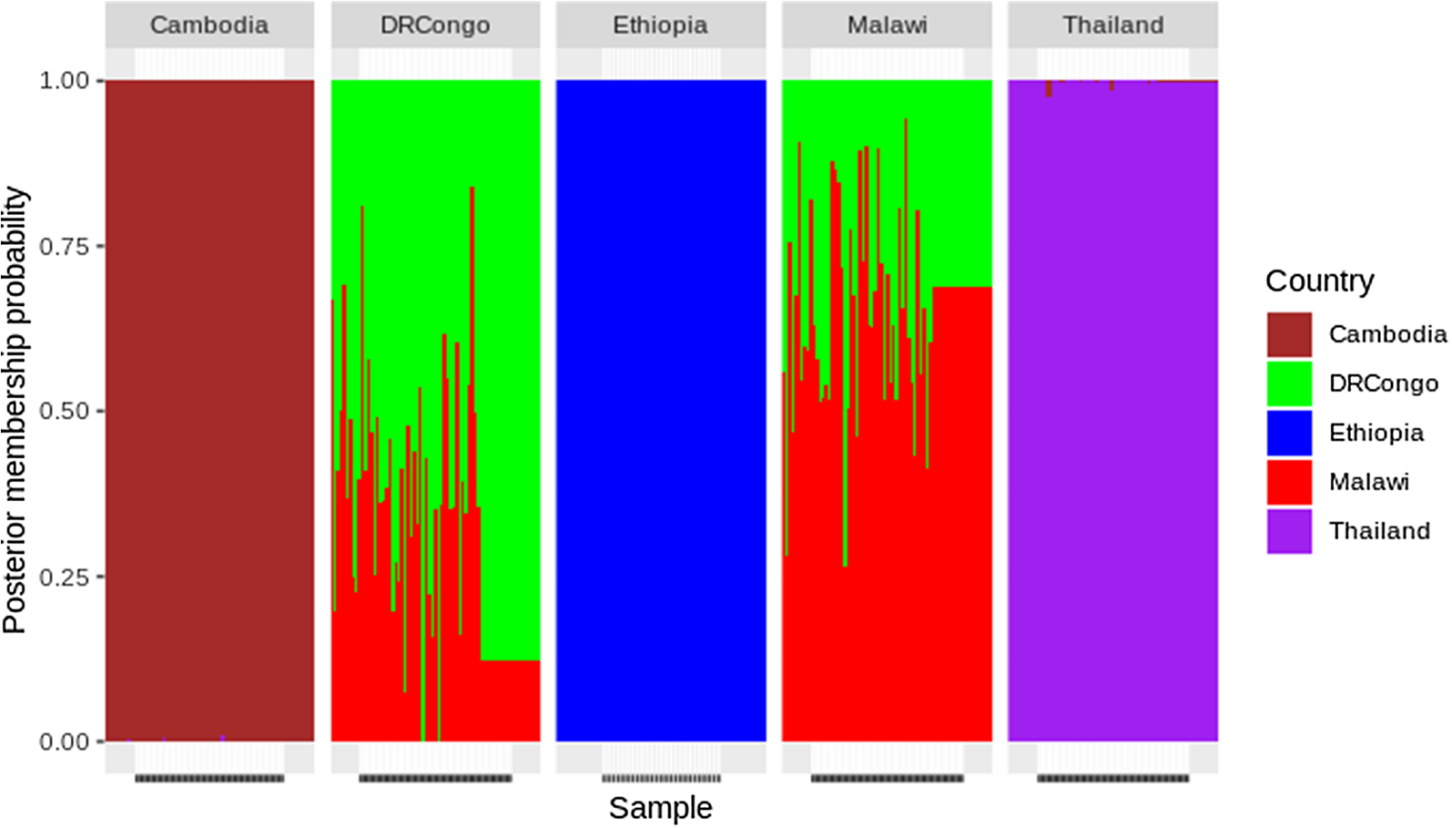
An admixture analysis: X-axis represent the assigned samples to country listed above the figure after modeling allele sharing probability, while Y-axis represent the membership probability of samples. Colour-coded country listed at the right of figure indicated the origin of *P. falciparum* sample collection. Analysis of admixture identified four major components based on an optimized cluster value of K = 5, and multiple subpopulations of *P. falciparum* were found in DR Congo and Malawi. There was a bidirectional gene flow between Malawian and Congolese *P. falciparum* populations

Analysis using PCA revealed the presence of four clear major population groups of isolates, which were coincident with their geographical origins (Fig. 2a–c). Similarly, the findings from admixture analysis were consistent with the PCA clustering. The isolates from the three regions were distinguished. This admixture analysis showed that four major components could be differentiated with a cluster value of K = 5. Multiple parasite subpopulations were observed in Malawi and DR Congo parasite populations suggestive of gene flow between these two populations (Fig. 3). There was no detectable gene flow between the isolates from Ethiopia and East African or Southeast Asia.

The clustering of Ethiopian *P. falciparum* isolates was consistent with the fixation index (F_ST_) values with or without correcting for sample size. The F_ST_ values of Ethiopian isolates versus those from the two other East African regions (DR Congo and Malawi) ranged from 0.08 to 0.09, while the F_ST_ value of Ethiopian *P. falciparum *versus the two southeast Asian regions (Thailand and Cambodia) was 0.18 (Table 2).

**Table 2:** Pairwise population divergence (measured by FST) among *P. falciparum* populations

Country	Cambodia	DR Congo	Ethiopia	Malawi	Thailand
Cambodia	0	0.16	0.18	0.17	0.06
DR Congo	0.16	0	0.08	0.02	0.16
Ethiopia	0.18	0.08	0	0.09	0.18
Malawi	0.17	0.02	0.09	0	0.17
Thailand	0.06	0.16	0.18	0.17	0

Ethiopian *P. falciparum* highly diverged from both Southeast Asian and East African *P. falciparum* populations

### Signatures of selection in the *P. falciparum* isolates

The Ethiopian isolates had the average Tajima’s D value of 0.18 across the entire genome (one sample t-test, p < 2 × 10^–16^). 1,450 genes had at least one SNP with Tajima D value > 1 of which 125 genes had at least five SNPs with Tajima D values > 1 of which 125 genes had at least five SNPs with Tajima D values > 1 (Additional file 4). These genes include *apical membrane antigen-1* (*ama1), erythrocyte binding antigen-175 (eba175), merozoites surface protein-1 (msp1), thrombospondin-related anonymous protein (trap), duffy binding like merozoites surface protein (dblmsp)*, and *cytoadherence linked asexual protein 2 (clag2)*, that were previously reported for the balancing selection [24, 33].

The standardized integrated haplotype homozygosity score (IHS) was applied to investigate genome-wide evidence for recent positive directional selection due to drug pressure, immune impact, or other mechanisms. Using |IHS| score of > 2.5 (top 1% of the expected distribution) as a threshold for hits, 36 genes with at least one SNP that could be under significant positive selection were identified, and out of these, 15 genes had at least two SNPs (Table 3).

**Table 3:** Genes with at least two SNPs that had a recent positive directional selection in *P. falciparum* of Ethiopia, identified using the integrated haplotype score at a significance threshold of P < 0.01

Chromosomes	Number of SNPs	Genes name or ID	Product description
1	9	PF3D7_0104100	Conserved *Plasmodium* membrane protein, unknown function
1	3	PF3D7_0113600	Surface-associated interspersed protein 1.2 (SURFIN 1.2)
4	2	PF3D7_0424300	Erythrocyte binding antigen-165
4	14	SURF4.2	Surface-associated interspersed protein 4.2 (SURFIN 4.2)
4	5	PF3D7_0425200	*Plasmodium* exported protein (hyp15), unknown function
4	4	PF3D7_0425250	*Plasmodium* exported protein (PHIST), unknown function
7	5	PF3D7_0713900	Conserved *Plasmodium* protein, unknown function
7	3	CRMP2	Cysteine repeat modular protein 2
8	3	CLAG8	Cytoadherence linked asexual protein 8
10	2	PF3D7_1004800	ADP/ATP carrier protein, putative
12	2	PF3D7_1201400	*Plasmodium* exported protein, unknown function
13	7	PF3D7_1301800	Surface-associated interspersed protein 13.1 (SURFIN 13.1)
13	3	PF3D7_1308400	Conserved *Plasmodium* protein, unknown function
14	2	PF3D7_1434500	Dynein-related AAA-type ATPase, putative
14	2	PF3D7_1477500	*Plasmodium* exported protein (PHISTb), unknown function

SNPs and ID stand for single nucleotide polymorphisms and gene identification numbers, respectively

Thirteen (13) out of the above 15 genes under positive directional selection showed both positive balancing and directional selections (Table 4) and these genes include the vaccine candidate gene *SURF4.2* on chromosome 4 and *CLAG8* (cytoadherence-linked asexual protein 8) on chromosome 8 [34]. Interestingly, attempts to detect selection signals in drug resistance genes, such as *Pfcrt*, *Pfmdr1*, *Pfdhfr*, and *Pfdhps* were unsuccessful. The reason could be that IHS may not be suitable for detecting positive selection for those SNPs that have reached or are near fixation in the local *P. falciparum* population [32].

**Table 4:** Genes under both recent positive directional selection and positive balancing selections in Ethiopian *P. falciparum* populations

Chromosomes	Gene name/ID	Product description
1	PF3D7_0104100	Conserved *Plasmodium* membrane protein, unknown function
1	PF3D7_0113600	Surface-associated interspersed protein 1.2 (SURFIN 1.2)
4	PF3D7_0424300	Erythrocyte binding antigen-165, pseudogene
4	SURF4.2	Surface-associated interspersed protein 4.2 (SURFIN 4.2)
4	PF3D7_0425200	*Plasmodium* exported protein (hyp15), unknown function
4	PF3D7_0425250	*Plasmodium* exported protein (PHIST), unknown function
7	PF3D7_0713900	Conserved Plasmodium protein, unknown function
8	CLAG8	Cytoadherence linked asexual protein 8
10	PF3D7_1004800	ADP/ATP carrier protein, putative
12	PF3D7_1201400	*Plasmodium* exported protein, unknown function
13	PF3D7_1301800	Surface-associated interspersed protein 13.1 (SURFIN 13.1)
13	PF3D7_1308400	Conserved Plasmodium protein, unknown function
14	PF3D7_1434500	Dynein-related AAA-type ATPase, putative

ID stands for a gene identification number

### Prevalence of mutations conferring anti-malarial drug resistance in *P. falciparum*

Table 5 shows inter-population differences in the prevalence of drug resistance genes observed among the *P. falciparum* global datasets analysed. In tandem with previous studies [20, 35] that suggest temporal differences in the geographical distribution of anti-malarial drug resistance mutations, in this study CQ-resistance alleles (*Pfcrt-K76T*, *Pfcrt-A220S*, and *Pfcrt-Q271E*) were fixed in Ethiopia, Cambodia, and Thailand, regions where malaria transmission rates are comparably low. In contrast, the prevalence of these same alleles was 0% in Malawi and ranged from 66 to 72% in DR Congo.

**Table 5:** Drug resistance-conferring allele’s frequency across the 5 *P. falciparum* populations

Genes	Chromosome	Position	Mutation site	Ethiopia	Cambodia	DR Congo	Malawi	Thailand
*DHFR*	4	748,577	*I164L*	0	0.5	0	0	0.84
*DHFR*	4	748,410	*S108N*	0	1	1	1	1
*DHFR*	4	748,262	*C59R*	0.86	1	0.86	0.99	1
*DHFR*	4	748,239	*N51I*	1	0.95	1	1	0.95
*MDR1*	5	961,625	*D1246Y*	0	0	0.17	0	0
*MDR1*	5	958,145	*N86Y*	0.14	0	0.48	0.03	0
*MDR1*	5	961,566	*F1226Y*	0	0.04	0	0	0.59
*MDR1*	5	958,440	*Y184F*	1	0.58	0.32	0.35	0.06
*CRT*	7	405,600	*I356T*	0	0.52	0.27	0	1
*CRT*	7	405,362	*N326S*	0.98	0.51	0	0	1
*CRT*	7	405,838	*R371I*	0	0.8	0.71	0	1
*CRT*	7	404,407	*A220S*	1	1	0.663	0	1
*CRT*	7	403,625	*K76T*	1	1	0.66	0	1
*CRT*	7	404,836	*Q271E*	1	1	0.7143	0	1
*DHPS*	8	549,685	*G437A*	0.08	0.13	0.08	0.01	0
*DHPS*	8	549,995	*K540N*	0	0.4	0	0	0.03
*DHPS*	8	549,681	*S436A*	0	0.2	0.11	0.02	0.17
*DHPS*	8	550,117	*A581G*	0.02	0.4	0.03	0.02	0.82
*K13*	13	1,726,432	*K189T*	0.2	0	0.17	0.13	0
*K13*	13	1,725,259	*C580Y*	0	0.36	0	0	0.26

Similarly, drug resistance mutations in *Pfmdr1* (*Pfmdr1*-*N86Y* and *Pfmdr1*-*Y184F*) were also variable among populations. For instance, the Ethiopian parasite population showed the presence of 14% *Pfmdr1-N86Y* and 100% *Pfmdr1*-*Y184F* gene mutations, whereas *Pfmdr1-N86Y*was detected in 48% of DR Congo isolates and in 3% of Malawi’s. Also, the *Pfmdr1*-Y184F drug resistance marker was detected in 58% of the *P. falciparum* population in Cambodia, 32% in DR Congo, 35% in Malawi, and 6% in Thailand’s parasite isolates.

Sulfadoxine/pyrimethamine drug resistance mutations were also present in *Pfdhfr* and *Pfdhps* genes in all analysed *P. falciparum* populations. The major pyrimethamine resistance-conferring alleles, such as *Pfdhfr*-*N51I* and *Pfdhfr*-*C59R*, were also identified in all parasite populations with fixed or near fixation in frequency. *Pfdhfr-S108N* was fixed in other *P. falciparum* populations, except in Ethiopia. The variable prevalence of drug resistance-conferring alleles were also observed in *Pfdhps* (*Pfdhps*-*S436A*, *Pfdhps*-*G437A*, *Pfdhps-K540N*, and *Pfdhps-A581G*), for the parent drug sulfadoxine resistance.

In terms of artemisinin resistance, the African population-specific *Pfk13*-*K189T* mutation was observed in Ethiopia (in 20% of the samples), DR Congo (17%), and Malawi (13%). This mutation was previously identified in African *P. falciparum* populations [20, 35]. As previously reported [8], the validated and most characterized artemisinin resistance-conferring mutation *PfK13*-*C580Y* was identified in Cambodia (36% of the samples) as well as in Thailand (26%), but not in Africa.

## Discussion

The transmission dynamic coupled with the unique history, ecology, and demography of Ethiopia raises interest in the genetics of its parasite population. High-resolution whole-genome SNP data was used to analyse *P. falciparum* parasite genetic diversity in the central region of Ethiopia and compared with similar parasite data from mainland Africa (DR Congo and Malawi) and Southeast Asian parasites, from Cambodia and Thailand. In this analysis, similar MAF across all five parasite populations with over-representation of low frequency (< 5%) variants was observed as previously reported [19, 21]. Interestingly, mean F_WS_ values were significantly higher in the Ethiopian parasite isolates as compared to the other African populations, but not the Southeast Asian parasite populations. F_WS_ is a genome-wide metric that averages heterozygosity across the genome in comparison with heterozygosity within the local parasite population [21]. Hence, it is a measure of within-host diversity of infections that helps to gauge the potential for inbreeding (or outcrossing). The higher F_WS_ values (> 0.95) in Ethiopia (*P. falciparum* prevalence of 0.02) [36] and East Asian infections is underscored by the low malaria transmission rates in these settings which supports a higher inbreeding and clonal propagation of infections (Fig. 1; Additional file 3). Unlike the other East African countries (DR Congo and Malawi) where transmission intensities are higher [20, 35], and there was a good distribution of F_WS_ values with the majority of infections being polyclonal with high potential for outcrossing (Fig. 1). These findings are supported by similar studies that link lower F_WS_ values to in west African where transmission is high [18]. However, of note, is the possibility for high F_WS_ values to occur in areas of high transmission intensity if *P. falciparum* circulates in a geographically isolated community which limits the chance of outcrossing with other genetically distinct *P. falciparum* parasites as observed in the previous study [21].

An analysis of parasite population structure within and between continents revealed a higher degree of population structure between Ethiopian parasites and other East African parasites and between Southeast Asia and East Africa. However, neither PCA (Fig. 2) nor admixture analysis (Fig. 3) could resolve parasite populations in DR Congo and Malawi. These observations are corroborated by several studies that report regional and inter-continental level structure in global *P. falciparum* parasite populations [21]. However, the separation of Ethiopian parasites from the two East African populations is worth noting. Notwithstanding the increased human mobility between Addis Ababa and the rest of Africa, particularly East Africa, there remain important barriers to gene flow between parasite populations in central Ethiopia and the rest of the sub-region. Indeed, one possible factors that severely limit gene flow between Ethiopia and its neighbours is the local malaria transmission intensity as a function of poor vectoral capacity determined by the ecological landscape (highlands).

Against the backdrop of this unique eco-epidemiology of *P. falciparum* malaria in Ethiopia, Tajima D and IHS was used to explore the mechanisms of natural selection in the country. However, identification of many antigenic genes under balancing selection with Tajima D value greater than one were observed in Ethiopia. These genes included known vaccine candidates, such as *ama1*, *trap*, *msp1*, *eba175*, and *clag2* (Additional file 4), which were previously identified in different populations that vary in transmission intensity [24, 33, 37], to be under balancing selection. Besides, 15 genes under positive directional selection by IHS were identified, which includes *SURFIN* and *PHIST* families previously suggested to be targets of immunity [24]. It can be hypothesized that the low seasonal transmission in Ethiopia maintains significant immune selection pressure on the infection reservoir than drug pressure due to clinical malaria. Therefore, the candidate vaccine antigen loci under balancing selection may be largely due to immune modulation and not positive adaptive selection influenced by drug pressure. This is supported by our failure to detect selection signatures in known drug target genes such as *Pfcrt*, *Pfmdr1*, *Pfdhfr*, *Pfdhps*, and *Pfkelch-13*. The ability of IHS to detect selection in these drug resistance genes in Ethiopia may be because the frequency of polymorphisms in these loci are either fixed or near fixation in the Ethiopian population (Table 5). These findings are supported by a previous study in Ethiopia which showed that the CQ-resistant haplotype (CVIET) was fixed [7]. The continued use of CQ in Ethiopia for the treatment of *P. vivax* malaria may account for the high prevalence of CQ resistant markers. Also, *Pfmdr 1* mutations have been demonstrated to mediate AL resistance. Therefore, the high prevalence of *Pfmdr 1* mutations may signal poor efficacy of AL as the first treatment for *P. falciparum* malaria in Ethiopia. Variable prevalences of CQ-resistant polymorphisms were observed only in DR Congo and not in Malawi, evidence that supports the complete reversal of CQ susceptibility in Malawi as reported by Ochola et al*.* [20].

Undoubtedly, artemisinin resistance has taken root in Southeast Asia. Despite 36% and 26% prevalence of *PfKelch13-C580Y* mutation in Cambodia and Thailand samples, respectively, no validated *PfKelch13* mutation was found in the African samples. However, an uncharacterized *Pfkelch13* mutation (*PfK13*-*K189T)* found at prevalence > 10% in all the African datasets and previously reported in other studies [35], may be important, but its role in artemisinin resistance is unknown. One study [8] reported that mutation in *Pfkelch13* at amino acid positions less than 441 may not play any role in mediating artemisinin resistance. A validated *Pfkelch13-R561H* mutation for artemisinin resistance was recently reported in other East African *P. falciparum* populations [9].

## Conclusion

Overall, this study reveals the presence of a comparably low genetic diversity of *P. falciparum* parasites in Ethiopia. The majority of infections were of low complexity, demonstrated significant population structure with Ethiopian parasites diverged from parasite populations within the sub-region. Based on the analysis made it is suggested the presence of limited gene flow between parasite populations in the East African sub-region and Ethiopia. More importantly, apparent balancing selection in antigenic loci known to be targets of immunity and adaptive positive selection in *SURFIN* and *PHIST* gene families that are potential vaccine antigens. Though selection analysis did not pick up any adaptive mutations in known drug-resistant genes, CQ-resistance *Pfcrt-K76T* genotype seems fixed in Ethiopia like the wild-type genotype (*K*) in Malawi. In this analysis no *PfKelch13* validated mutations were reported in Ethiopia, DR Congo, and Malawi except a *PfK13*-*K189T* African specific uncharacterized mutation. Further molecular studies involving deeper sampling of Ethiopian parasite populations are essential to understand the genetic diversity, gene flow, and temporal evolution of drug resistance loci within Ethiopia. Furthermore, such findings can be used to support national malaria control decision-making for optimal impact in further reducing malaria transmission in Ethiopia.

## Supplementary Information

**Additional file 1.** Summary of sequence reads and ENA accession number per isolate included in this study.

**Additional file 2: Figure S1.** Distribution of the number of SNPs across all analyzed genes and their respective SNPs type distribution. **Figure S2.** Minor allele frequency distribution by population. SNPs are binned into 10 equal sizes of 0.05. In all parasite populations, there is an overabundance of low-frequency SNPs (MAF < 5%).

**Additional file 3.** F_WS_ values of samples in *P. falciparum* populations analyzed in this study.

**Additional file 4.** Genes that had ≥ 5 SNPs and a Tajima D of ≥ 1 that are defined as genes under positive balancing selection.

## Data Availability

Datasets generated and/or analysed during the study are available through the MalariaGEN Pf3K Project. The *P. falciparum* genome sequences used in this study are available in the ENA and SRA databases (see Additional file 1 for accession numbers).

## Abbreviations

ACT: Artemisinin-based combination therapy
AL: Artemether–lumefantrine
ART: Artemisinin
CQ: Chloroquine
FWS: Within infection diversity fixation index
FST: Population differentiation fixation index
HW: Heterozygosity within infection
HS: Heterozygosity within a local population
IHS: Standardized integrated haplotype score
IRS: Indoor Residual Spraying
ITN: Insecticide-treated nets
*Pfcrt*: *Plasmodium falciparum chloroquine*
*Pfdhfr*: *Plasmodium falciparum dihydrofolatereductase*
*Pfdhps*: *Plasmodium falciparum pteroate synthase*
*PfK13*: *Plasmodium falciparum kelch-13*
SNP: Single nucleotide polymorphisms
SP: Sulfadoxine/pyrimethamine
