# Supplementary material for: Genomic analysis reveals independent evolution of Plasmodium falciparum populations in Ethiopia
Source: Malar J. 2021 Mar 4;20:129. doi: 10.1186/s12936-021-03660-y (PMC7934276; doi:10.1186/s12936-021-03660-y)

**Additional file 2**

**Figure S1**. Distribution of the number of SNPs across all analyzed genes and their respective SNPs type distribution. **A)** Distribution of the number of genes (Cambodia; N=4134, DR Congo; N=4722, Ethiopia; N=3370, Malawi; N=4789, Thailand; N=4029) containing at least one SNP across the genome. **B)** Distribution of polymorphic SNP marker types in each P. falciparum population. NONSYN: Non-synonymous, SYN: Synonymous, INTER: Intergenic, INTRON: Intronic and INTRA: Other intragenic SNP respectively. Color coded by country.


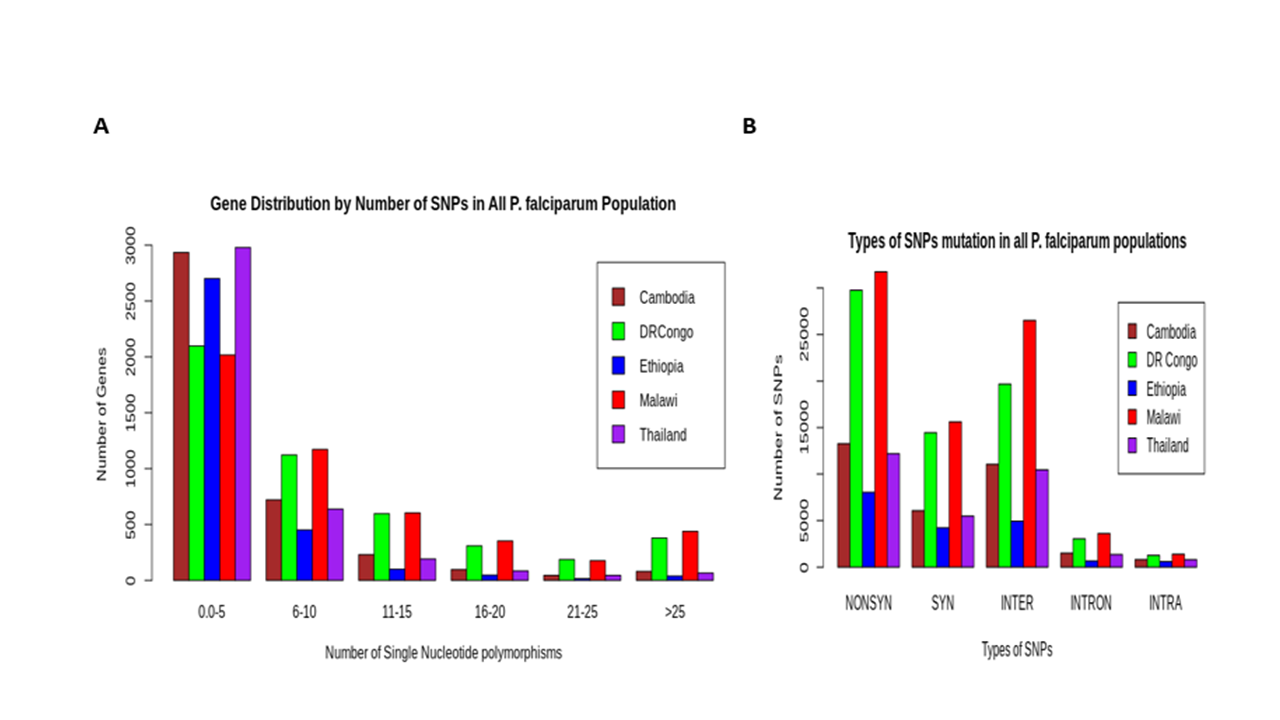


**Figure S2**. Minor allele frequency Distribution by population. SNPs are binned into 10 equal sizes of 0.05. In all parasite populations, there is an overabundance of low-frequency SNPs (MAF < 5%).


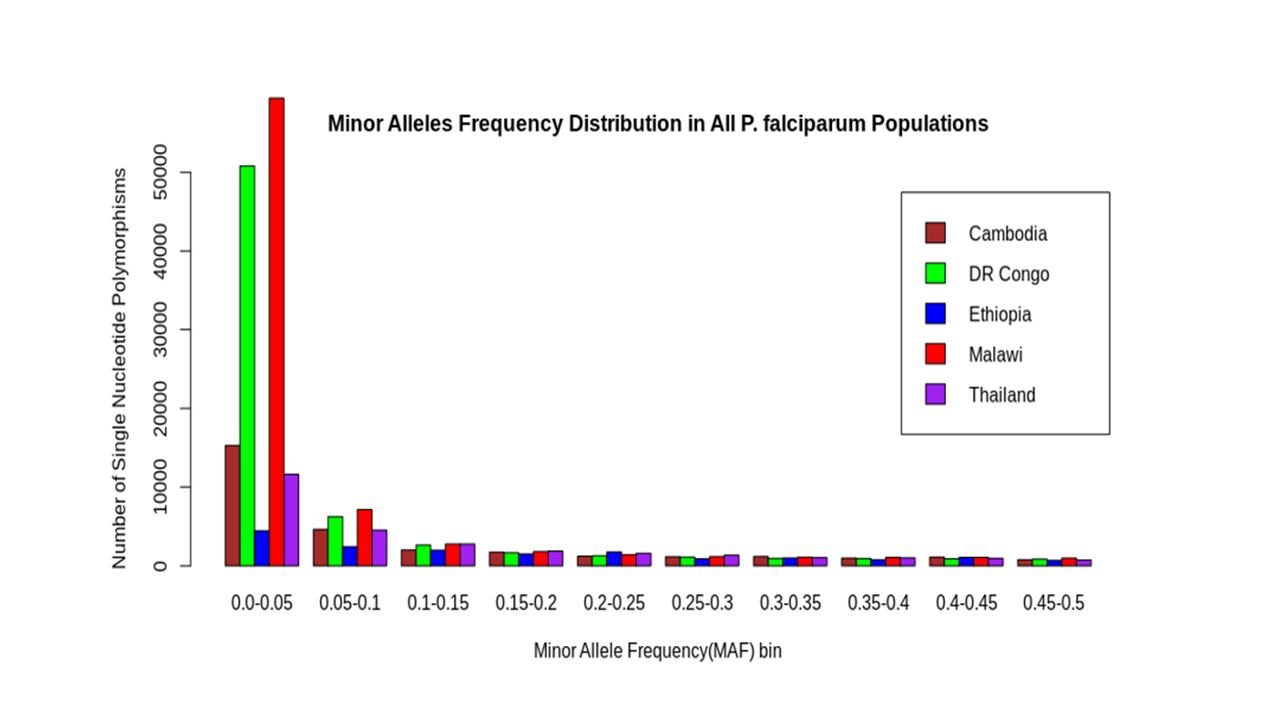

Supplement: Supplementary file 2 — Additional file 2: Figure S1. Distribution of the number of SNPs across all analyzed genes and their respective SNPs type distribution. Figure S2. Minor allele frequency distribution by population. SNPs are binned into 10 equal sizes of 0.05. In all parasite populations, there is an overabundance of low-frequency SNPs (MAF < 5%). [file 12936_2021_3660_MOESM2_ESM.doc]
